# MB-DECTNet: a model-based unrolling network for accurate 3D dual-energy CT reconstruction from clinically acquired helical scans

**DOI:** 10.1088/1361-6560/ad00fb

**Published:** 2023-12-08

**Authors:** Tao Ge, Rui Liao, Maria Medrano, David G Politte, Jeffrey F Williamson, Joseph A O’Sullivan

**Affiliations:** 1 Washington University in St. Louis, Saint Louis, MO 63130, United States of America

**Keywords:** CT reconstruction, dual-energy CT, deep learning, model-based learning, material decomposition, deep unrolling

## Abstract

*Objective.* Over the past several decades, dual-energy CT (DECT) imaging has seen significant advancements due to its ability to distinguish between materials. DECT statistical iterative reconstruction (SIR) has exhibited potential for noise reduction and enhanced accuracy. However, its slow convergence and substantial computational demands render the elapsed time for 3D DECT SIR often clinically unacceptable. The objective of this study is to accelerate 3D DECT SIR while maintaining subpercentage or near-subpercentage accuracy. *Approach.* We incorporate DECT SIR into a deep-learning model-based unrolling network for 3D DECT reconstruction (MB-DECTNet), which can be trained end-to-end. This deep learning-based approach is designed to learn shortcuts between initial conditions and the stationary points of iterative algorithms while preserving the unbiased estimation property of model-based algorithms. MB-DECTNet comprises multiple stacked update blocks, each containing a data consistency layer (DC) and a spatial mixer layer, with the DC layer functioning as a one-step update from any traditional iterative algorithm. *Main results.* The quantitative results indicate that our proposed MB-DECTNet surpasses both the traditional image-domain technique (MB-DECTNet reduces average bias by a factor of 10) and a pure deep learning method (MB-DECTNet reduces average bias by a factor of 8.8), offering the potential for accurate attenuation coefficient estimation, akin to traditional statistical algorithms, but with considerably reduced computational costs. This approach achieves 0.13% bias and 1.92% mean absolute error and reconstructs a full image of a head in less than 12 min. Additionally, we show that the MB-DECTNet output can serve as an initializer for DECT SIR, leading to further improvements in results. *Significance.* This study presents a model-based deep unrolling network for accurate 3D DECT reconstruction, achieving subpercentage error in estimating virtual monoenergetic images for a full head at 60 and 150 keV in 30 min, representing a 40-fold speedup compared to traditional approaches. These findings have significant implications for accelerating DECT SIR and making it more clinically feasible.

## 1. Introduction

Dual-energy computed tomography (DECT) methods employ data gathered at two distinct x-ray energy levels, producing more comprehensive and quantifiable outcomes, and have been widely applied in various medical applications such as automated bone extraction in CT angiography, blood pool imaging, and virtual non-contrast-enhanced imaging (McCollough *et al* 2015). In the realm of radiotherapy (van Elmpt *et al* 2016, Kruis 2022), DECT has substantially enhanced the precision of electron density (Tsunoo *et al* 2008), effective atomic number (Goodsitt *et al* 2011, Bonnin *et al* 2014, Hua *et al* 2018, Schaeffer *et al* 2021), and proton-stopping power imaging (Han *et al* 2016, Taasti *et al* 2016, Zhang *et al* 2018, Chang *et al* 2022, Peters *et al* 2022, Yang *et al* 2023). The latest advancements in DECT include the implementation of statistical iterative reconstruction (SIR) algorithms, which reduce noise while improving resolution and accuracy. A novel SIR algorithm has been created that functions on disparate-energy raw-data sinograms and attains sub-percentage uncertainty in imaging proton-stopping power maps (Medrano *et al* 2022, Ge *et al* 2023).

**Figure 1.**
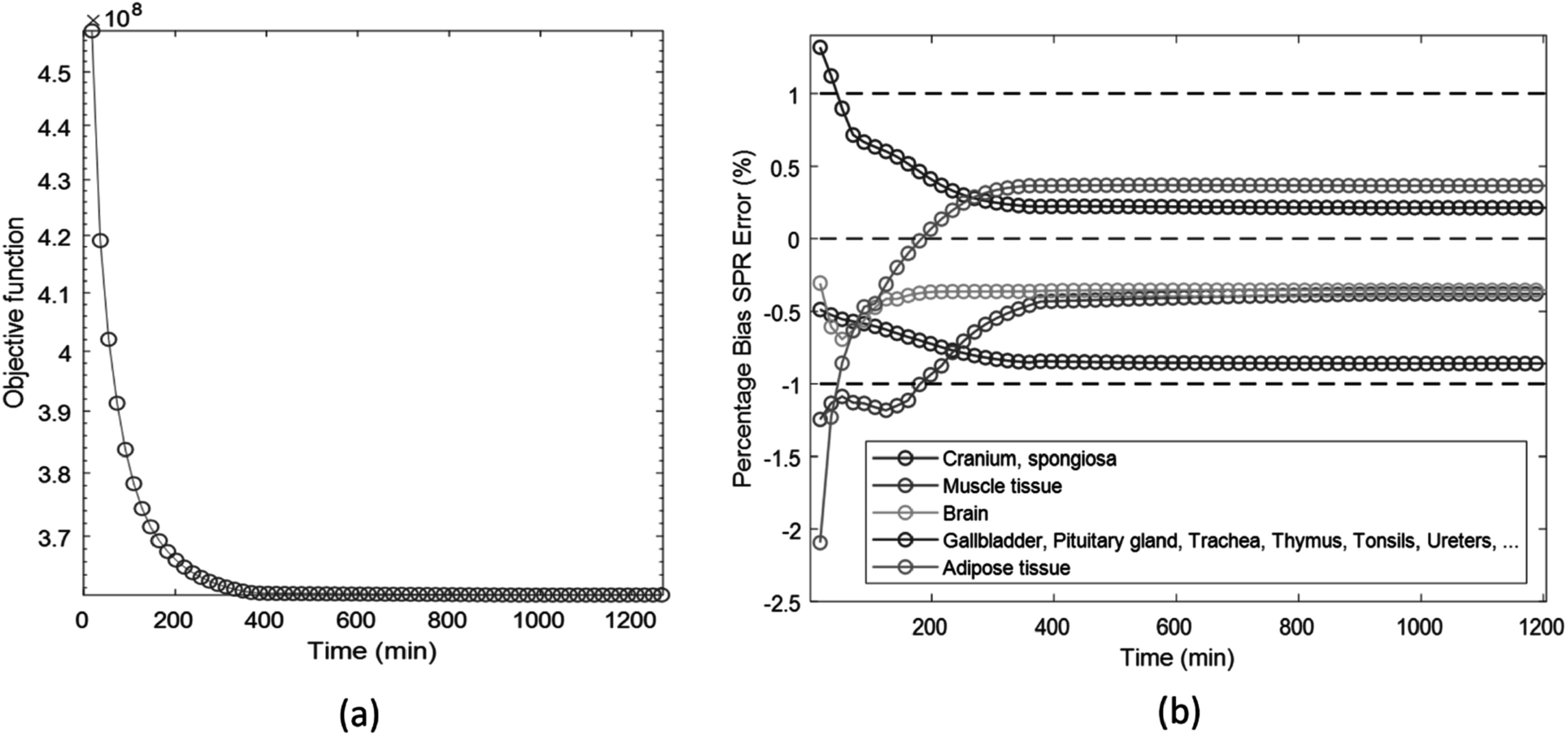
(a) Plot of the objective function value versus elapsed time of DEAM. (b) Plot of the SPR estimation bias versus elapsed time of DEAM.

However, CT scanner geometries have become increasingly complex and large in scale. However CT scanner geometries have become increasingly complex and large in scale. Consequently, DECT SIRs can be time-intensive when reconstructing three-dimensional (3D) image volumes from helical scans, even when utilizing acceleration techniques such as multi-GPU acceleration (Liu *et al* 2017, Mitra *et al* 2017) and switching ordered-subsets (Erdogan and Fessler 1999, Degirmenci *et al* 2015). This arises because a surrogate function is often used to account for the nonlinearity of the polychromatic forward model, which results in a large gap between the surrogate used to compute iterative updates and the real objective function, resulting in a sublinear rate of convergence. Additionally, as CT scanner system geometries become larger, evaluation of the system operator becomes increasingly expensive computationally. Another more minor contributing factor is that DECT algorithms involve two different spectra, doubling the system operations per iteration compared to single-energy CT (SECT). The slow convergence rate and substantial computational demands of projections present challenges in obtaining accurate DECT results within a clinically acceptable time frame. To exemplify this issue, figure 1 demonstrates the time-to-convergence of a DECT SIR, dual-energy alternating minimization (DEAM), when reconstructing an anthropomorphic phantom with a 100 mm thickness from a simulated measurement involving 12 rotations (O’Sullivan and Benac 2007). Despite the relatively small geometry, DEAM requires approximately 400 min to converge.

Deep-learning (DL) techniques have been extensively investigated for image denoising and reconstruction in medical imaging. Traditional DL uses a deep neural network to learn and model complex patterns and relationships within the training data. The convolutional neural network (CNN) has gained prominence due to its ability to effectively identify local patterns and spatial correlations. The U-Net architecture (Ronneberger *et al* 2015), initially designed for image segmentation, has gained popularity in the field of image restoration and reconstruction due to its encoder–decoder configuration, which facilitates a larger receptive field compared to conventional deep CNNs, making it easy to capture both global and local contexts. However, studies have reported that pure deep-learning techniques may be unstable in medical image reconstruction, particularly with regard to the response of small perturbations in the image domain (Antun *et al* 2020).

To address instability, researchers have merged deep learning with physical models to build deep model-based networks. Variable splitting algorithms such as ADMM (Boyd *et al* 2011) and proximal gradient descent (Nitanda 2014) form the basis for combining traditional model-based iterative algorithms with neural networks. The penalty-minimizing step in these algorithms is replaced by a DL-based denoiser to achieve better regularization performance (Venkatakrishnan *et al* 2013, Romano *et al* 2016, Sun *et al* 2019, Zhang *et al* 2021, Liu *et al* 2022). More relevant to our setting, an iterative neural network for material decomposition has been proposed in (Li *et al* 2023) that plugs a learnable prior to block coordinate descent for DECT reconstruction. This method achieves approximately 2% of mean square error and requires at least 100 iterations to converge.

The deep unrolled network is another model-based DL structure that simulates iterative algorithms using a series of learnable networks (Yang *et al* 2016, Jin *et al* 2017, Aggarwal *et al* 2019, Chun *et al* 2020, Liu *et al* 2021, Ramzi *et al* 2022). This type of network is trained end-to-end using a semi-supervised setup, minimizing the discrepancy against the target image, as well as the inconsistency against the measurement through the forward model. A total variation prior unrolling method (Zhang *et al* 2023) was proposed to unroll the Chambolle-Pock algorithm for two-dimensional single-energy CT reconstruction. The training data consisted of simulated normal dose measurements from clinical low-dose CT images. This network with 10 unrolling iterations achieves 37.8 PSNR in reconstructing the out-of-sample simulated normal dose sinograms.

In this study, we introduce a DL approach for DECT reconstruction aimed at reducing the elapsed time while retaining estimation accuracy, and provide proof of principal testing. This is achieved by integrating DECT SIR into a deep learning model-based unrolled network for DECT reconstruction (MB-DECTNet). This deep learning-based method is trained to learn the shortcuts between the initial conditions and the stationary points of iterative algorithms while preserving the unbiased estimation property of model-based algorithms. We replace the data consistency layer in the deep unfolding with DECT update directions. Each block within the network constitutes a DECT SIR iteration, with the network determining the step size and image prior. We quantitatively compare the proposed MB-DECTNet with a purely DL-based approach employing a U-Net structure. Furthermore, we demonstrate that the MB-DECTNet output can be utilized to initialize DECT SIR, yielding further efficiency improvements. To the best of our knowledge, our study reports the first development of an unrolled network specifically designed for statistical DECT reconstruction.

## 2. Materials and method

### 2.1. Statistical dual-energy CT reconstruction

In this study, we modeled the linear attenuation coefficient (LAC) as a sum of products of spatial functions times energy functions as

\begin{eqnarray*}\mu (x,E)=\displaystyle \sum _{i}{\mu }_{i}(E){c}_{i}(x),\end{eqnarray*}

where $x\in {{\mathbb{R}}}^{3}$ denotes the spatial location in the image domain, *E* denotes the energy index, *μ*(*x*, *E*) denotes the LAC value at location *x* and energy *E*, *i* indexes the basis component, *μ*_*i*_(*E*) denotes the LAC of the selected material, and *c*_*i*_(*x*) denotes the basis component weights that need to be reconstructed.

In DECT statistical iterative reconstruction (SIR), the stationary point is reached by minimizing the objective function with respect to the target image ***c*** = {*c*_1_, *c*_2_} as

\begin{eqnarray*}\hat{{\boldsymbol{c}}}=\arg \,{\min }_{{\boldsymbol{c}}}{ \mathcal D }({\boldsymbol{c}},{\boldsymbol{d}})+{ \mathcal R }({\boldsymbol{c}}),\end{eqnarray*}

where ${ \mathcal D }$ denotes the data-fidelity term, ${ \mathcal R }$ denotes the penalty term, ***d*** = {*d*_*L*_, *d*_*H*_} denotes the set of measurements acquired at low- and high-kVp spectra.

The choice of data fidelity term depends on the assumed nature of the measured noise. If Poisson noise is assumed, I-divergence may be employed (O’Sullivan and Benac 2007, Long and Fessler 2014, Chen *et al* 2016), whereas a weighted least square is utilized if Gaussian noise is assumed (Huh and Fessler 2009, Xu *et al* 2009, Zhang *et al* 2014). Consequently, the target image *c* undergoes iterative updates based on the penalized selected fidelity function.

Compared to image-domain techniques, SIR reconstructs more accurate images with higher resolution and less uncertainty (Zhang *et al* 2018, Medrano *et al* 2022). However, DECT SIRs are usually computationally intensive due to the large system operator and the low convergence rate caused by the surrogate function that addresses the nonlinearity of the forward model.

### 2.2. MB-DECTNet

**Figure 2.**
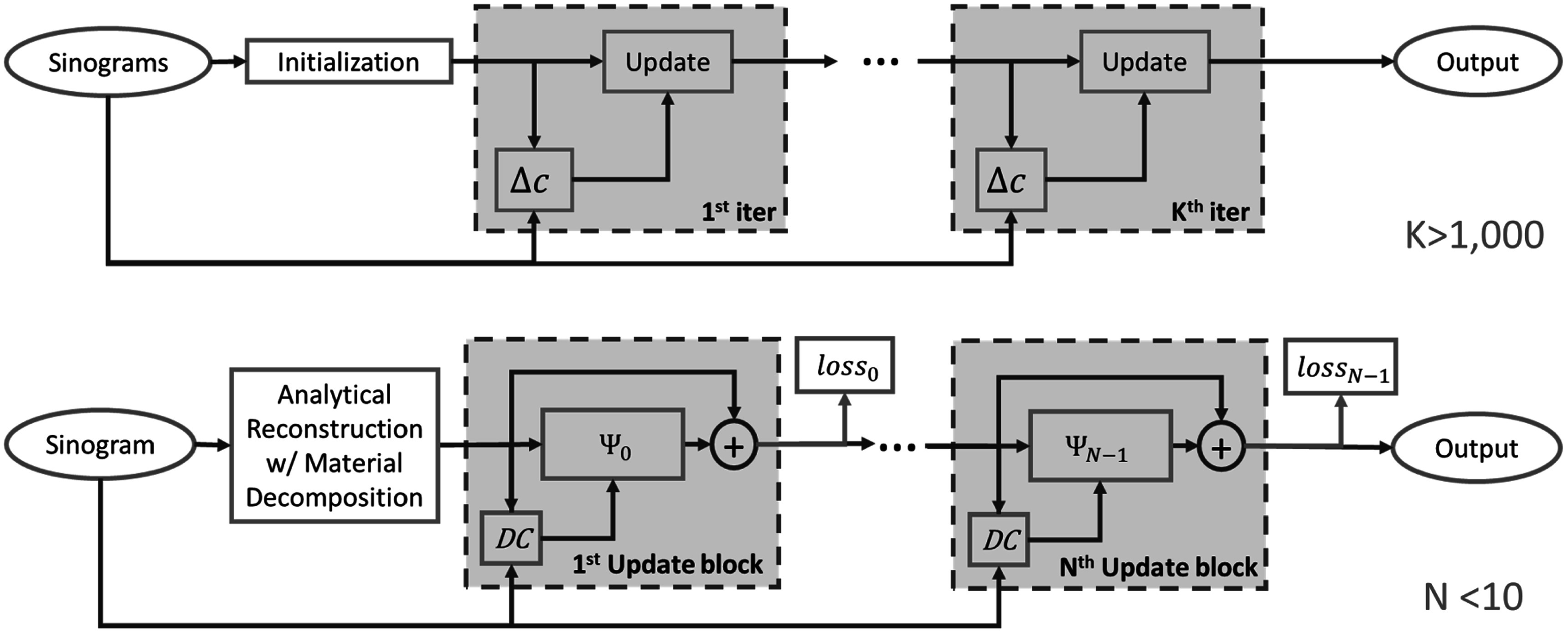
Upper figure: flowchart of model-based iterative reconstructions (MBIR). Lower figure: flowchart of MB-DECTNet. Each blue dashed box denotes an update block, which mimics an iteration in DECT SIR, and the orange boxes denote the evaluation of loss functions, through which the gradient will be backpropagated.

The framework of the traditional DECT SIR is shown in the upper part of figure 2, where (2) is solved by iteratively updating the target image based on the update direction. The update step for such algorithms can be written as

\begin{eqnarray*}{{\boldsymbol{c}}}^{k}={{\boldsymbol{c}}}^{k-1}+{\gamma }^{k-1}{\mathrm{\Delta }}{{\boldsymbol{c}}}^{k-1},\end{eqnarray*}

where *k* denotes the iteration number, Δ ***c*** ^*k*−1^ denotes the update direction, and *γ* ^*k*−1^ is the step size. This kind of algorithm usually requires thousands of iterations to converge.

Our model-based deep unrolling network (MB-DECTNet) replaces the update step with a neural network, and Δ*c* with the *data-consistency* (DC) layer, which ensures that the unrolled network, (lower picture of figure 2) is model-based. Here Ψ_*k*_ denotes the *k*th deep image updater. MB-DECTNet consists of several *update blocks* that are analogous to iterations in SIR. Intuitively, these networks learn a more aggressive, high-dimensional step size, as well as a denoiser from the training set. With this model-based unrolled network, we are trying to get the stationary point in just a few iterations.

**Figure 3.**
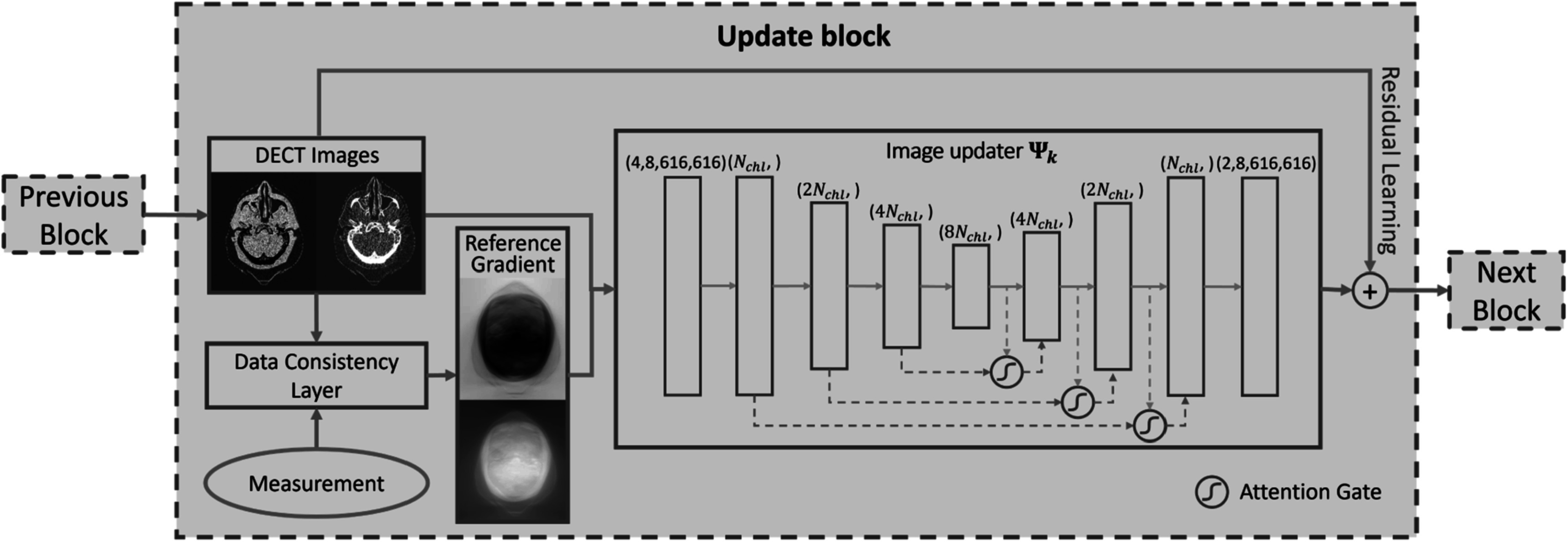
Structure of an update block. The number of channels is increased from 4 to *N*_chl_, 2N_chl_, etc *N*_chl_ can be adjusted based on GPU memory usage. *Reference Gradient* denotes the output of the DC layer that provides physical-based gradient information for two basis components, *c*_1_ and *c*_2_.

The structure of the update block is shown in figure 3. The DC layer takes the image from the previous block and the transmission data as the inputs to derive the update direction, which quantifies the inconsistency between the current estimate and the measurement. The update direction can vary with respect to the algorithm type. For gradient descent optimizers, it is defined as the simple gradient of the objective function with respect to the previous estimate ***c*** ^*k*−1^,

\begin{eqnarray*}{\mathscr{DC}}({{\boldsymbol{c}}}^{k-1},d)={\left.\displaystyle \frac{\partial { \mathcal D }({\boldsymbol{c}},{\boldsymbol{d}})}{\partial {\boldsymbol{c}}}\right|}_{{\boldsymbol{c}}={{\boldsymbol{c}}}^{k-1}},\end{eqnarray*}

or as the stationary point of the surrogate function minus the previous estimate ***c*** ^*k*−1^ when SIR minimizes a surrogate of the objective function

\begin{eqnarray*}{\mathscr{DC}}({{\boldsymbol{c}}}^{k-1},d)=\left\{\arg \mathop{\min }\limits_{{\boldsymbol{c}}}\tilde{{ \mathcal D }}\{{\boldsymbol{c}},{\boldsymbol{d}},{{\boldsymbol{c}}}^{k-1}\}\right\}-{{\boldsymbol{c}}}^{k-1},\end{eqnarray*}

where *k* denotes the index of the current block, and $\tilde{{ \mathcal D }}$ denotes the surrogate function of the data fidelity term at ***c*** ^*k*−1^. Since the update block can be regarded as a denoiser, an explicit penalty term ${ \mathcal R }(c)$ in (2) is not necessary.

The DECT image and DC output are stacked along the channel dimension as the input to the deep learning-based image updater Ψ_*k*_. The subsequent estimate is evaluated by the pointwise summation of the previous estimate and the network output. With this residual learning strategy, MB-DECTNet is able to focus on the inconsistency of the estimate against the training target (He *et al* 2015, Zhang *et al* 2016), making training more efficient. To sum up, in the proposed model-based unrolling network, (3) in DECT SIR is rewritten as

\begin{eqnarray*}{{\boldsymbol{c}}}^{k}={{\boldsymbol{c}}}^{k-1}+{{\mathrm{\Psi }}}_{k}\left({\mathtt{stack}}({\mathscr{DC}}({{\boldsymbol{c}}}^{k-1},d),{{\boldsymbol{c}}}^{k-1})\right).\end{eqnarray*}

In this work, we adapted a shrunken U-Net as the deep learning-based image updater. The channel number of the first layer *N*_chl_ varies according to the memory footprint of each update block. With the increase in the depth, the number of layers in the encoder part increased to 2N_chl_, 4N_chl_, and 8N_chl_, and then decreased by a factor of 2 after each concatenation operation in the decoder part.

Attention gates (Oktay *et al* 2018) were deployed before the concatenation operations in the shrunk UNet. This implementation enables the image updater to capture salient features and learn to focus on image structures exhibiting a higher level of inconsistency with the stationary point. In the attention gate, the input from the skip connection is scaled by an attention coefficient. The attention coefficient is evaluated by combining the input itself with the gating signal from a coarser scale, followed by a ReLU layer, a linear layer, and a soft activation function.

In order to stabilize and speed up the training process, our network is trained under the supervision of the weighted sum of a set of intermediate loss functions. For the sake of simplicity, we rewrite RHS in (6) as ${{\mathrm{\Gamma }}}_{{\theta }_{k}}({{\boldsymbol{c}}}^{k-1})$. The final loss function is

\begin{eqnarray*}\arg \,{\min }_{\{{\theta }_{0}\ldots {\theta }_{n}\}}\displaystyle \sum _{n=0}^{N-1}{{\mathrm{w}}}_{t,n}\cdot { \mathcal L }\left({{\boldsymbol{c}}}_{\mathrm{truth}},{{\mathrm{\Gamma }}}_{{\theta }_{n}}\circ {{\mathrm{\Gamma }}}_{{\theta }_{n-1}}...\circ {{\mathrm{\Gamma }}}_{{\theta }_{0}}({{\boldsymbol{c}}}_{\mathrm{init}})\right),\end{eqnarray*}

where *w*_*t*,*n*_ ∈ [0, 1] denotes the scalar that controls the back-propagation weight, *N* denotes the number of update blocks, *t* denotes the index of the current training epoch, and {*θ*_0_, *θ*_1_} denotes the set of trainable parameters in all update blocks. ◦denotes the function composition, and ${\mathfrak{L}}$ denotes the customized loss function for the dual-energy BVM model

\begin{eqnarray*}{ \mathcal L }({{\boldsymbol{c}}}_{\mathrm{truth}},{{\boldsymbol{c}}}_{\mathrm{est}})=\displaystyle \sum _{i}\parallel {c}_{i,\mathrm{truth}}-{c}_{i,\mathrm{est}}{\parallel }_{2}^{2}+\displaystyle \sum _{E}{\psi }_{L}(E)\parallel \displaystyle \sum _{i}{\mu }_{i}(E){c}_{i,\mathrm{truth}}-\displaystyle \sum _{i}{\mu }_{i}(E){c}_{i,\mathrm{est}}{\parallel }_{2}^{2},\end{eqnarray*}

where ∥ · ∥_2_ is the *ℓ*_2_ norm, and *ψ*_*L*_(*E*) denotes the lower-energy spectrum at energy *E*. The second physics-based loss term is introduced to account for the correlation between *c*_1_ and *c*_2_.

### 2.3. Incorporate DEAM into MB-DECTNet

In this study, we used dual-energy alternating minimization (DEAM) to demonstrate the performance of MB-DECTNet. DEAM is a jointly statistical image reconstruction technique that maximizes the log-likelihood of Poisson random variables with realizations ***d*** and estimated mean polychromatic sinogram ***g*** (***c***) = {*g*_*L*_(***c***), *g*_*H*_(***c***)} (O’Sullivan and Benac 2007).

Maximizing the Poisson likelihood is equivalent to minimizing the I-divergence

\begin{eqnarray*}{ \mathcal I }({\boldsymbol{d}}\parallel {\boldsymbol{g}}({\boldsymbol{c}}))=\displaystyle \sum _{j}\displaystyle \sum _{y}{d}_{j}(y)\mathrm{log}\displaystyle \frac{{d}_{j}(y)}{{g}_{j}(y:{\boldsymbol{c}})}-{d}_{j}(y)+{g}_{j}(y:{\boldsymbol{c}}),\end{eqnarray*}

where *j* = {*L*, *H*}, and *g*_*j*_(*y*: ***c***) is given by the polychromatic forward model as

\begin{eqnarray*}{g}_{j}(y:{\boldsymbol{c}})=\int {I}_{0,j}(E)\exp \left(-\int h(x,y)\displaystyle \sum _{i}{c}_{i}(x){\mu }_{i}(E){dx}\right){dE}+{\xi }_{j}(y),\end{eqnarray*}

where $y\in {{\mathbb{R}}}^{3}$ denotes the location in the measurement space, *h*(*x*, *y*) denotes the system operator, and *I*_0_(*y*, *E*) denotes the mean photon count in the absence of the object, including the spectrum and bowtie filter. In the presence of substantial scatter, a scatter term *ξ*_*j*_(*y*) may be introduced. Derivation of an ideal scatter model remains an active research topic (Liu *et al* 2021, Maier *et al* 2019, Medrano *et al* 2022).

Decoupling the I-divergence in energy, spatial, and basis domain yields an analytical solution to the surrogate function (refer to the appendix for derivation and step size *Z*_*i*_(*x*)):

\begin{eqnarray*}{c}_{i}^{k+1}(x)={c}_{i}^{k}(x)-\displaystyle \frac{1}{{Z}_{i}(x)}\mathrm{log}\displaystyle \frac{{\sum }_{j}{\sum }_{y}{\sum }_{E}{\mu }_{i}(E)h(x,y){p}_{j}^{k}(y,E)}{{\sum }_{j}{\sum }_{y}{\sum }_{E}{\mu }_{i}(E)h(x,y){q}_{j}^{k}(y,E)},\end{eqnarray*}

where

\begin{eqnarray*}{q}_{j}^{k}(y,E)={I}_{0,j}\exp \left(-\displaystyle \sum _{x}h(x,y)\displaystyle \sum _{i}{\mu }_{i}(E){c}_{i}^{k-1}(x)\right)\end{eqnarray*}

\begin{eqnarray*}{p}_{j}^{k}(y,E)={q}_{j}^{k}(y,E)\displaystyle \frac{{d}_{j}(y)}{{\sum }_{E^{\prime} }{q}_{j}^{k}(y,E^{\prime} )}.\end{eqnarray*}

Therefore, for DEAM, the DC layer in MB-DECTNet is given by the log ratio between the forward projection of the attenuation-weighted measured sinogram and the forward projection of the attenuation-weighted mean sinogram that are summed over measurement index *j* and energy *E*, and weighted by LACs of the selected materials, formulated as

\begin{eqnarray*}{\mathscr{DC}}({{\boldsymbol{c}}}^{k-1},d)=\left[\begin{array}{c}-\displaystyle \frac{1}{{Z}_{1}(x)}\mathrm{log}\displaystyle \frac{{\sum }_{j}{\sum }_{y}{\sum }_{E}{\mu }_{1}(E)h(x,y){p}_{j}^{k}(y,E)}{{\sum }_{j}{\sum }_{y}{\sum }_{E}{\mu }_{1}(E)h(x,y){q}_{j}^{k}(y,E)}\\ -\displaystyle \frac{1}{{Z}_{2}(x)}\mathrm{log}\displaystyle \frac{{\sum }_{j}{\sum }_{y}{\sum }_{E}{\mu }_{2}(E)h(x,y){p}_{j}^{k}(y,E)}{{\sum }_{j}{\sum }_{y}{\sum }_{E}{\mu }_{2}(E)h(x,y){q}_{j}^{k}(y,E)}\end{array}\right].\end{eqnarray*}

### 2.4. Memory efficiency

The GPU memory footprint is a critical design issue for 3D unrolled networks due to the large size of the image and multiple unrolling blocks. Besides the shrunken UNet mentioned in the previous subsection, we partition the image volume into multiple small stacks consisting of 8 slices to reduce memory consumption. The transmission data corresponding to each image stack is also truncated accordingly. For the geometry used in this work, 8 slices correspond exactly to a gantry rotation of the scanner. While the term ‘slice’ typically has a different meaning in CT reconstruction, in the context of this paper, it refers to a collection of voxels that share the same z-position.

We used 4×NVIDIA Tesla V100 to train the model. Each V100 has 32 GB memory, which can only accommodate a single instance of the training data. Therefore, we employed the gradient accumulation technique to compensate for the small batch size used in our training process. Instead of updating the model parameters after each singleton batch, we accumulated the gradients across multiple batches and updated the model every four samples to simulate a training process with a batch size of 4. Moreover, group normalization (Wu and He 2018) is utilized rather than the widely-used batch normalization in order to increase the accuracy of statistics estimation. These two techniques help reduce the memory requirements during training and improve the speed and stability of training, especially when GPU memory is limited.

### 2.5. Margin effect

**Figure 4.**
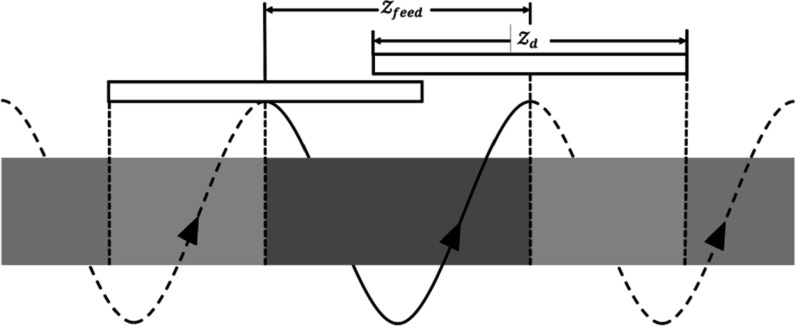
Schematic diagram of the margin effect. The solid spiral curve denotes the current gantry rotation. The bright orange rectangle denotes the select slices to be reconstructed, while the light orange rectangles denote the slices that are not reconstructed but where the corresponding sinogram data still contribute to the reconstructed images (bright orange). Flat boxes denote the detector array at different gantry steps.

In helical CT reconstruction, it is crucial to account for the margin effect, especially when a thin slice is reconstructed. As depicted in figure 4, the image slices corresponding to a single gantry rotation (highlighted in orange) are determined by the central row of the first and last views, where ${{ \mathcal Z }}_{d}$ represents the collimation width, and ${{ \mathcal Z }}_{\mathrm{feed}}$ denotes the bed travel distance along the *z*-direction per rotation. However, light orange segments outside the ROI will still influence the data acquired, and vice versa. Therefore, if the DC layer only evaluates the update direction based on the brighter orange area, the update direction will be much larger than expected to compensate for the absence of the light orange area. Thus, it is essential to pad the input image volume along the *z*-direction to ensure accurate results from the DC layer. Note that the diagram assumes parallel x-ray beams due to the large ratio between the source-to-detector distance and ${{ \mathcal Z }}_{d}$, which does not accurately represent the actual geometry in our implementations.

Furthermore, each slice is not reconstructed from the same number of views. Let *z* be the distance between the centers of the first and an arbitrary slice. The number of gantry steps contributing to a slice can be calculated as

\begin{eqnarray*}{n}_{\mathrm{vps}}(z)=\left\{\begin{array}{cc}{n}_{\mathrm{vpr}}\left(\frac{{{ \mathcal Z }}_{d}}{2{{ \mathcal Z }}_{\mathrm{feed}}}+\frac{z}{{{ \mathcal Z }}_{\mathrm{feed}}}\right) &amp; z< {{ \mathcal Z }}_{\mathrm{feed}}-\frac{{{ \mathcal Z }}_{d}}{2}\\ {n}_{\mathrm{vpr}}\left(\frac{{{ \mathcal Z }}_{d}}{2{{ \mathcal Z }}_{\mathrm{feed}}}+\frac{{{ \mathcal Z }}_{\mathrm{feed}}-1-z}{{{ \mathcal Z }}_{\mathrm{feed}}}\right) &amp; z> \frac{{{ \mathcal Z }}_{d}}{2}-1\\ {n}_{\mathrm{vpr}} &amp; \mathrm{else},\end{array}\right.\end{eqnarray*}

where *n*_vpr_ denotes the number of views per rotation.

The reference gradient for peripheral slices will be updated based on fewer views compared to the gradient for central slices. This implies that the peripheral slices of the reconstructed image may exhibit higher error margins than the central slices. To mitigate this issue, only the central slices of the MB-DECTNet output were utilized to evaluate the performance of the proposed method, and the contribution of peripheral slices to the loss function was down-weighted. Ideally, we want to extract the slices that correspond to as many as possible views. In the clinical setup, ${{ \mathcal Z }}_{\mathrm{feed}}$ is 8.304 mm and ${{ \mathcal Z }}_{d}$ is 12 mm. Therefore, the slice index must satisfy $2.2\leqslant \tilde{z}\leqslant 4.8$ to ensure the image slice is reconstructed by a full rotation of views.

**Figure 5.**
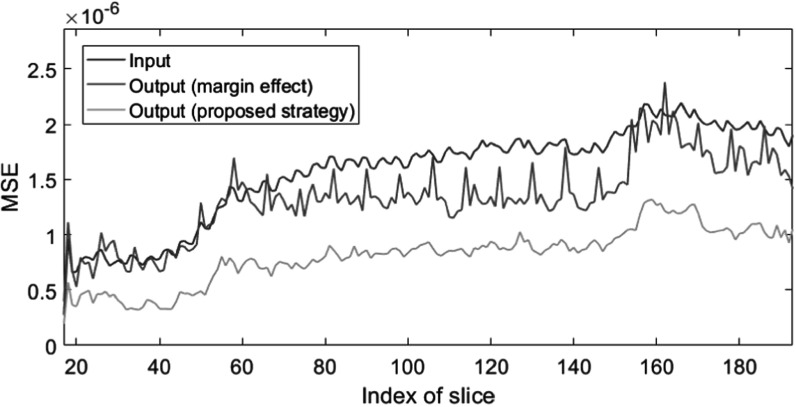
Slice-wise MSE of the neural network outputs versus slice index. Output (margin effect) denotes the test output suffering from the margin effect, and output (proposed strategy) denotes the test output with the central-slice strategy.

Figure 5 shows the plots of estimation error by slices along the *z*-direction. A periodic pattern can be seen in the MSE plot of output with the margin effect. The model-based network with the proposed strategy significantly outperforms the same network that is trained and tested based on all the slices of the samples.

### 2.6. Gradient of DC layer

During the training process, the weight of the neural network is updated based on the gradient of the loss function with respect to the corresponding parameter. Following the chain rule, this gradient can be propagated from the loss function to the foremost layer, a technique commonly referred to as back-propagation.

The Jacobian of the DC layer can be expressed as

\begin{eqnarray*}\begin{array}{l}\displaystyle \frac{\partial }{\partial {c}_{i^{\prime} }(x^{\prime} )}{\mathscr{DC}}{\left({\boldsymbol{c}},d\right)}_{(i,x)}={\left[\displaystyle \sum _{j}\displaystyle \sum _{y}\displaystyle \sum _{E}\displaystyle \frac{\partial {\mathscr{DC}}({\boldsymbol{c}},d)}{\partial {q}_{j}(y,E)}\displaystyle \frac{\partial {q}_{j}(y,E)}{\partial {\boldsymbol{c}}}\right]}_{(x,i),(x^{\prime} ,i^{\prime} )}\\ \quad =-\displaystyle \frac{1}{{Z}_{i}(x)}\displaystyle \sum _{j^{\prime} }\displaystyle \sum _{y^{\prime} }\displaystyle \sum _{E^{\prime} }\displaystyle \frac{\partial }{\partial {q}_{j^{\prime} }(y^{\prime} ,E^{\prime} )}\mathrm{log}\\ \quad \cdot \displaystyle \frac{{\sum }_{j}{\sum }_{y}{\sum }_{E}{\mu }_{i}(E)h(x,y){q}_{j}(y,E)\tfrac{{d}_{j}(y)}{{\sum }_{E^{\prime\prime} }q(y,E^{\prime\prime} )}}{{\sum }_{j}{\sum }_{y}{\sum }_{E}{\mu }_{i}(E)h(x,y){q}_{j}(y,E)}\displaystyle \frac{\partial {q}_{j^{\prime} }(y^{\prime} ,E^{\prime} )}{\partial {c}_{i^{\prime} }(x^{\prime} )}\\ \quad =\displaystyle \frac{1}{{Z}_{i}(x){\left({\hat{b}}_{i}(x)\right)}^{2}}\displaystyle \sum _{j}\displaystyle \sum _{y}\displaystyle \sum _{E}{\mu }_{i}(E)h(x,y)\left({A}_{j}(y,E){\hat{b}}_{i}(x)-{\tilde{b}}_{i}(x)\right){q}_{j}(y,E){\mu }_{i^{\prime} }(E)h(x^{\prime} ,y)\\ \quad \approx -\displaystyle \frac{1}{{Z}_{i}(x){\hat{b}}_{i}(x)}\displaystyle \sum _{y}h(x,y)\left(\displaystyle \sum _{j}\displaystyle \sum _{E}{\mu }_{i}(E)\displaystyle \frac{{q}_{j}{\left(y,E\right)}^{2}}{{d}_{j}(y)}{\mu }_{i^{\prime} }(E)\right)h(x^{\prime} ,y)\end{array}\end{eqnarray*}

where

\begin{eqnarray*}{\hat{b}}_{i}(x)=\displaystyle \sum _{j}\displaystyle \sum _{y}\displaystyle \sum _{E}{\mu }_{i}(E)h(x,y){q}_{j}(y,E)\end{eqnarray*}

\begin{eqnarray*}{\tilde{b}}_{i}(x)=\displaystyle \sum _{j}\displaystyle \sum _{y}\displaystyle \sum _{E}{\mu }_{i}(E)h(x,y){p}_{j}(y,E)\end{eqnarray*}

\begin{eqnarray*}{A}_{j}(y,E)=\displaystyle \frac{{d}_{j}(y)\left({\sum }_{E^{\prime} }{q}_{j}(y,E^{\prime} )-{q}_{j}(y,E)\right)}{{\left({\sum }_{E^{\prime} }{q}_{j}(y,E^{\prime} )\right)}^{2}}.\end{eqnarray*}

**Figure 6.**
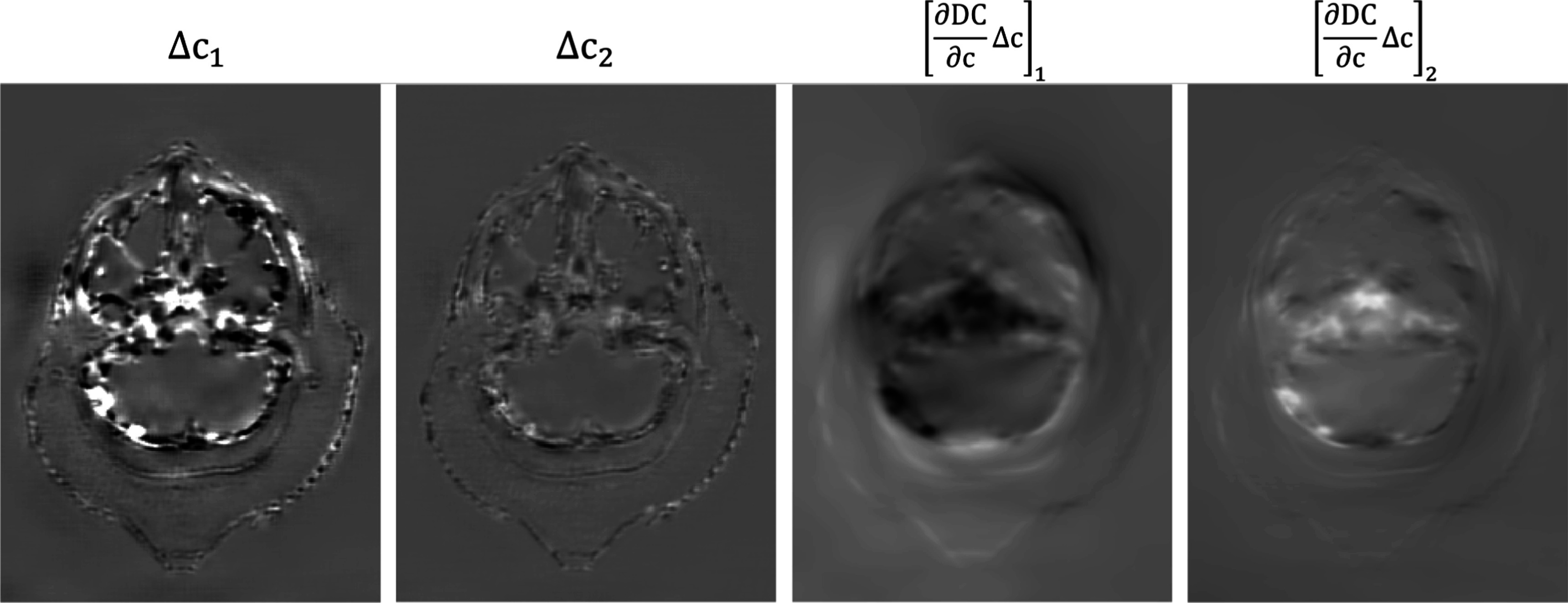
Backpropagated gradients before and after the DC layer. Display windows for the left two images: [−10, 10]. Display windows for the right two images: [−5 × 10^−6^, 5 × 10^−6^].

The last expression in (16) follows by assuming *g*_*j*_(*y*) ≈ *d*_*j*_(*y*) and *q*_*j*_(*y*, *E*) ≈ *p*_*j*_(*y*, *E*). Note that *q*_*j*_(*y*, *E*) and ${\hat{b}}_{i}(x)$ have been computed during forward propagation, so only 2 forward-projections and 2 back-projections are required for an update block per iteration. Figure 6 shows an example of the gradients before and after the DC layer.

During our experimentation, we observed that MB-DECTNet utilizing the exact DC gradient demonstrated a higher convergence rate than the MB-DECTNet implementation that relied on the zero DC gradient. However, it is noteworthy that both networks reached comparable levels of performance upon convergence.

### 2.7. Experiment description

In our study, we have performed a quantitative comparison between three MB-DECTNet variants and two benchmark methods: the prevalent image-domain decomposition (IDD) (Yan *et al* 2000) and a standalone deep learning technique (Pure Net). To comprehend the interplay between the model performance and the physical information incorporated, we introduced three MB-DECTNet variants with 1, 2, and 4 update blocks, referred to as MB-DECTNet-1, MB-DECTNet-2, and MB-DECTNet-4. Each variant comprises 1, 2, or 4 DC layers, representing increasing levels of synergy with the physical model. To maintain a balanced comparison, the channel count, *N*_chl_, in the MB-DECTNets was fine-tuned, ensuring roughly equivalent learnable parameters across models. For the Pure Net method, we employed the widely used U-Net structure (Ronneberger *et al* 2015) with both input and output channel numbers set to 2, representing a model devoid of physical knowledge.

To quantitatively compare the accuracy of various models, we plotted histograms of the estimated LAC errors at 30, 60, and 150 keV, where the error was calculated as

\begin{eqnarray*}\displaystyle \frac{{\mu }_{{est}}(x,E)-{\mu }_{{GT}}(x,E)}{{\mu }_{{GT}}(x,E)}\times 100 \% .\end{eqnarray*}

### 2.8. Training

To enhance training efficiency and promote training stability, we employed a pre-training strategy in this study. Specifically, we first trained the first block of the MB-DECTNet independently for 100 epochs using the ADAM optimizer with a starting learning rate of 10^−4^ and a decay rate of 0.8 every 10 epochs. During this stage, the input remained constant throughout epochs, and the reference update from the DC layer was reusable, resulting in a relatively quick training process. We then propagated the pre-trained weights to other blocks to initialize the complete unrolling network. Finally, we trained the entire network end-to-end for an additional 50 epochs, minimizing the loss function in equation (7) with a learning rate of 10^−4^ and a decay rate of 0.8 every 5 epochs. The loss weights *w*_*t*,*n*_ employed for MB-DECTNet with varying numbers of blocks are shown in table 1.

**Table 1.** Loss weights.

Epoch	*w*_*t*,*n*_ (1 block)	*w*_*t*,*n*_ (2 blocks)	*w*_*t*,*n*_ (4 blocks)
0 →10	[1.00 ]	[0.50, 1.00 ]	[0.30, 0.40, 0.50, 1.00 ]
11 →20	[1.00 ]	[0.30, 1.00 ]	[0.10, 0.10, 0.10, 1.00 ]
21 →∞	[1.00 ]	[0.01, 1.00 ]	[0.01, 0.01, 0.01, 1.00 ]

### 2.9. Data acquisition

In this study, we utilized images reconstructed by DEAM as the ground truth. The inputs are the image domain decomposition method with an iterative scheme (Yan *et al* 2000). The dual-energy measurements were obtained at 90 and 140 kVp using a Philips Brilliance Big Bore CT scanner located at the Alvin J Siteman Cancer Center in Washington University School of Medicine, following a helical protocol. In order to obtain the raw data necessary for our approach, beam hardening corrections in these measurements have been removed, which requires special agreement with the vendor. The number of patients available within the human subject protocol was limited. The detector array contains 16 rows and 816 channels, with a row spacing of 0.75 mm and channel spacing of 0.001 189 71 radians. Each rotation contains 1320 gantry steps with a *z*-direction feed of 8.304 mm.

These measurements were subsequently reconstructed through the application of a penalized DEAM within a motion-compensation framework (Ge *et al* 2023), with a sufficient number of iterations. Basis materials are 23% aqueous solution of CaCl_2_ and polystyrene (Williamson *et al* 2006). The size of the reconstructed head-neck image is 610 × 610 × 340, and the image resolution is 1 × 1 × 1.034 mm^3^. The penalty term is written as

\begin{eqnarray*}{ \mathcal R }({\boldsymbol{c}})=\displaystyle \sum _{x}\displaystyle \sum _{i}\displaystyle \sum _{x^{\prime} \in { \mathcal N }(x)}{w}_{R}(x,x^{\prime} )\psi \left({c}_{i}(x)-{c}_{i}(x^{\prime} )\right),\end{eqnarray*}

where ${ \mathcal N }(x)$ denotes the neighborhood of *x*, *w*_*R*_ denotes the weight addressing the distance between *x* and $x^{\prime} $. The derivation of the image update with the penalty of the form (21) is shown in the appendix. The potential function *ψ* is an element-wise convex symmetric smooth function:

\begin{eqnarray*}\psi (t)={\delta }^{2}\left(\displaystyle \frac{| t| }{\delta }-\mathrm{ln}\left(1+\displaystyle \frac{| t| }{\delta }\right)\right),\end{eqnarray*}

where *δ* controls the transition between approximate *ℓ*_2_-norm and approximate *ℓ*_1_-norm regions.

Each DEAM ground-truth image set took 20 h to converge on 4×NVIDIA V100 32 GB GPUs (400 iterations with 33 ordered subsets, followed by an additional 1000 iterations without ordered subsets).

The training set contains the images and measurements for 4 head-neck patients, one lung patient, and a customized head-sized phantom. The number of epochs was determined through a validation set containing out-of-sample lung patient data. Our test set contains 320 samples extracted from an out-of-sample head-neck patient scan.

Scans of patients treated with proton or photon therapy are acquired under IRB study NCT03403361. The scanned region of the head-neck patient extended from the top of the head to the base of the skull to model a typical central-nervous-system scan, and the scanned region of the lung patient extended from the base of the skull to the top of the pelvis to represent a typical lung scan.

The customized phantom has a cylindrical acrylic shell with a diameter of 215 mm and length of 20 mm, which is filled with water. Eight inserts are plastic bottles with a diameter of 31 mm and filled with liquid samples, including ethanol, propanol, butanol, and K_2_HPO_4_ solutions with different concentrations.

Each sample is the truncated portion of the spiral sinogram with dimensions 16 × 816 × 1320, which corresponds to a rotation of the scanner gantry. The image size of a sample was 610 × 610 × 8, with an image resolution of 1 × 1 × 1.034 mm^3^. In total, the training set contained 1280 samples.

## 3. Results

### 3.1. Quantitative results

**Figure 7.**
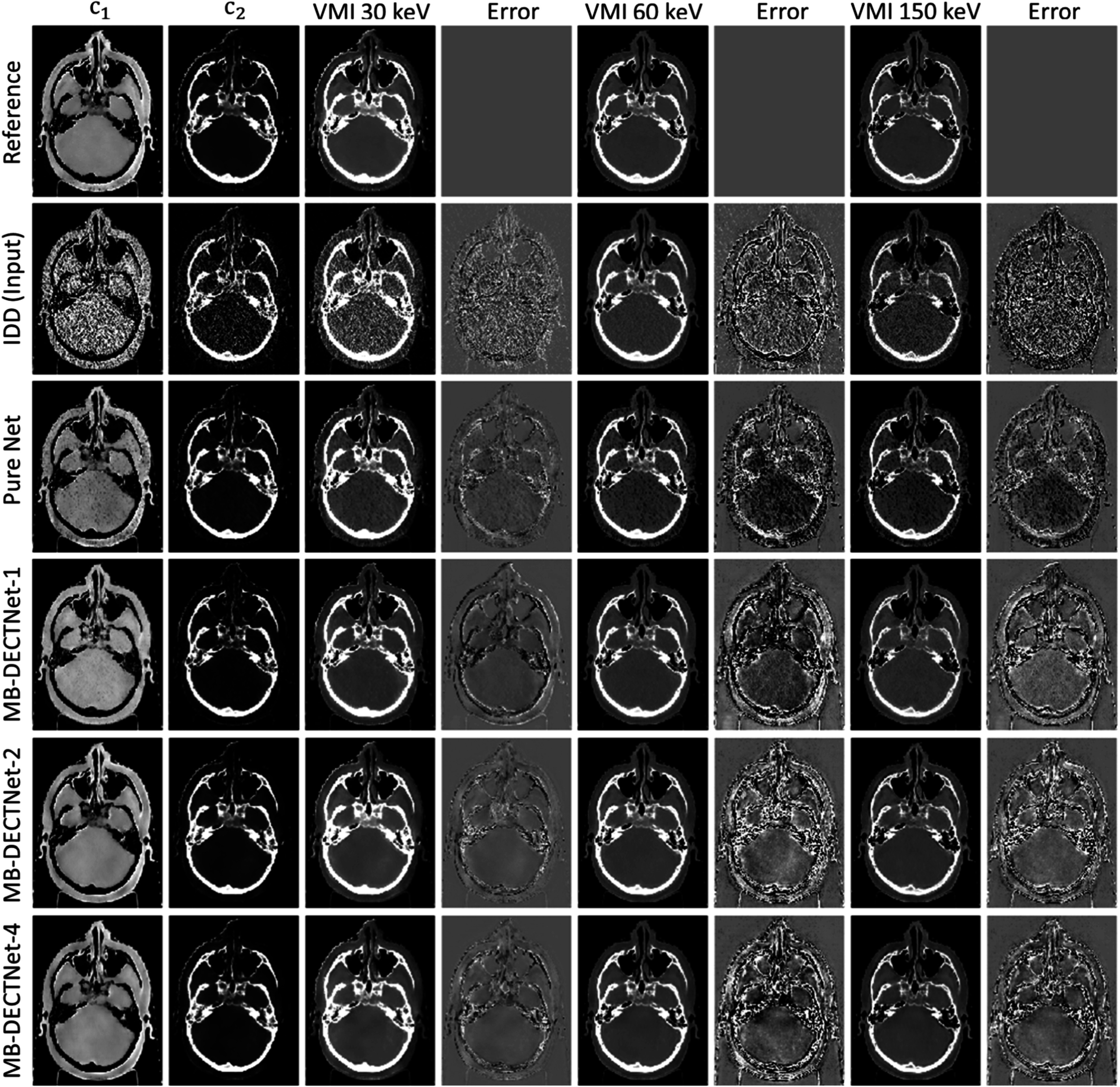
Images of the selected slice in the test dataset. IDD denotes the image-domain decomposition results, and Pure Net denotes the U-Net-based denoiser and bias corrector. MB-DECTNet-1, MB-DECTNet-2, and MB-DECTNet-4 are the proposed network with 1, 2, and 4 update blocks, respectively. Column (1), (2): *c*_1_, *c*_2_ (display window [0, 1.2]). Column (3), (5), (7): 30 keV VMI([0, 0.1]), 60 keV VMI ([0.01, 0.04]), and 150 keV VMI ([0.01, 0.025]). Column (4), (6), (8): error of the corresponding image against the reference at 30 keV ([−0.05, 0.05]), 60 keV ([−0.002, 0.002]), and 150 keV ([−0.002, 0.002]). The display window for VMI at 60 keV is [−514, 942] if it is interpreted as in HU.

Figure 7 illustrates the estimated component weights and virtual monoenergetic images (VMIs) of the selected slice in the test set. The IDD result has the highest noise level. The pure deep-learning model offers some noise reduction but exhibits an underestimation of the soft tissue in VMIs at 60 and 150 keV. MB-DECTNet with 1 update block (MB-DECTNet-1) offers better denoising capabilities compared to the pure network, but it tends to underestimate the LAC of bony tissues, particularly near the boundary between the skull and brain tissue. MB-DECTNet with 2 and 4 update blocks (MB-DECTNet-2 and MB-DECTNet-4) deliver the best visual performance and demonstrate the potential for reducing noise while preserving structural information. In inference mode, MB-DECTNet-2 and MB-DECTNet-4 generate a 610 × 610 × 340 image in roughly 352 and 700 s, which are 205 and 103 times shorter than the elapsed time of DEAM, respectively.

**Figure 8.**
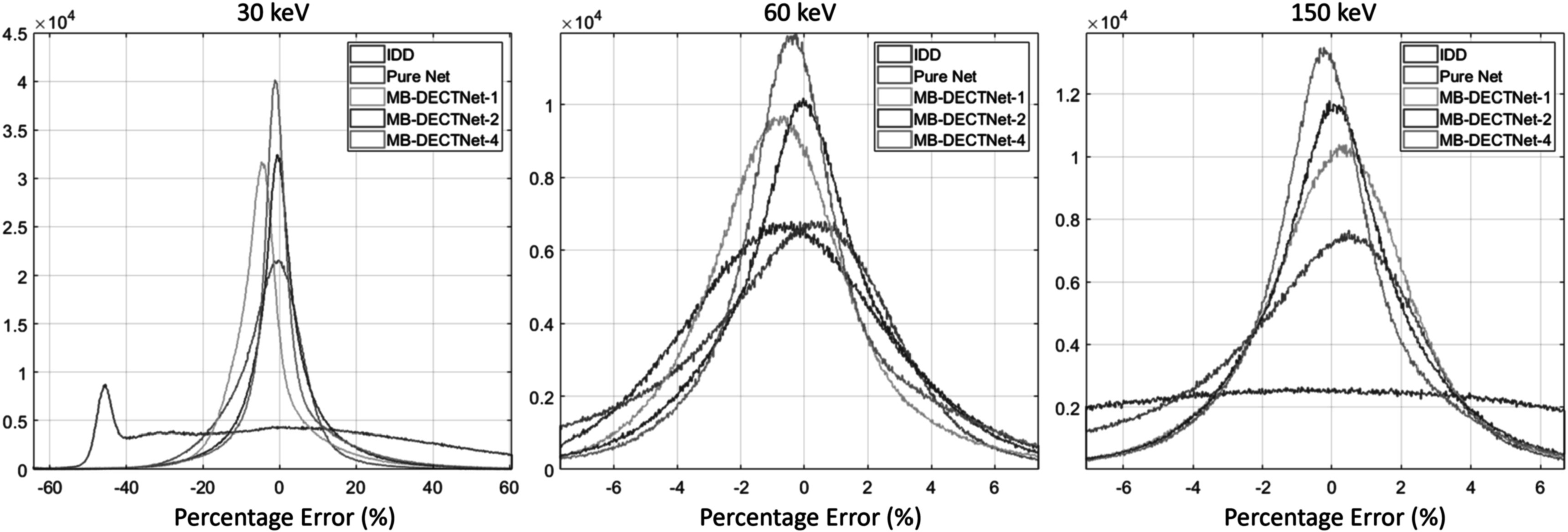
Histograms of percentage estimated LAC errors. A higher concentration of values near zero indicates better performance. The distribution tails were truncated to enhance clarity and legibility. The columns from left to right correspond to the estimated LAC at different energies.

The histograms of percentage errors are depicted in figure 8. The region of interest was limited to all soft and bony tissues (LAC threshold range [0.01, 0.05] at 60 keV) to avoid extremely small values being used as the denominator. The IDD estimation errors at 30 keV and 150 keV span a wide range, indicating a large variance in the corresponding VMIs. Additionally, the peak at −48% in the 30 keV plot suggests a significant estimation bias in IDD. All deep-learning models demonstrate the ability to correct bias and reduce noise in the initial image. As the pure network does not utilize data-consistency information, its performance is inferior to that of model-based networks. Among the model-based learning methods, MB-DECTNet-4 exhibits the most near-zero errors at the three selected energies, indicating that the proposed network with the most update blocks achieves the best performance.

Table 2 also reflects the same trend, where MB-DECTNet-4 emerges as the top performer across most evaluated metrics. MB-DECTNet-4 attains the lowest MAE and highest PSNR at 60 and 150 keV. The percentage bias of MB-DECTNet-2 at 150 keV is lower than that of MB-DECTNet-4, and their biases are comparable at 60 keV. Since the component weight estimates the LAC over a broad energy range, metrics at 60 and 150 keV may be insufficient to fully demonstrate the performance of the models. Hence, we utilized the original objective function of DECT SIR as an alternate performance measure that projects the estimates to the sinogram domain and directly compares them with the measurement. Among the deep-learning models, MB-DECTNet-4 achieves the lowest sinogram-domain objective function value.

**Table 2.** Performance evaluation using different image quality metrics. MB-Net-1, MB-Net-2, and MB-Net-4 are abbreviations for MB-DECTNet-1, MB-DECTNet-2, and MB-DECTNet-4, respectively. % Bias denotes the percentage bias, % MAE denotes the percentage mean absolute error, and PSNR denotes the peak-signal-to-noise ratio. These metrics are evaluated based on 60 and 150 keV mono-energetic images.

	% Bias 60 keV	% MAE 60 keV	PSNR 60 keV	% Bias 150 keV	% MAE 150 keV	PSNR 150 keV	Obj. func. (sinogram)
Reference	/	/	/	/	/	/	7.76E+9
IDD	−1.36	3.56	58.68	−3.13	7.68	56.67	4.35E+10
Pure Net	1.14	3.10	59.91	2.51	4.59	58.52	3.85E+10
MB-Net-1	−0.86	2.62	61.00	0.27	1.94	65.69	2.36E+10
MB-Net-2	**0.13**	2.13	62.30	**0.06**	1.92	66.48	1.41E+10
MB-Net-4	**0.13**	**1.92**	**62.82**	−0.25	**1.72**	**66.90**	**1.22E+10**

A noticeable disparity can be observed between the objective function of the reference image and the MB-DECTNet-4 estimate. However, this discrepancy does not necessarily imply suboptimal performance by MB-DECTNet-4. As commonly acknowledged, achieving a neural network output with precisely zero background is challenging. In the calculation of the objective function, the nonzero background values for air will be projected and accumulated in the sinogram domain, and undergo nonlinear amplification through the exponentiation term in the forward model. These background errors can be mitigated by post-processing.

### 3.2. Initialize DECT SIR using MB-DECTNet

The previous subsection demonstrated the potential of MB-DECTNet to substitute for model-based statistical algorithms for DECT reconstruction. However, it can be seen that due to the limited number of accessible unpreprocessed clinical measurements in the training set, the estimated image by MB-DECTNet did not meet the situation where highly accurate reconstruction results with the subpercentage error were desired.

**Figure 9.**
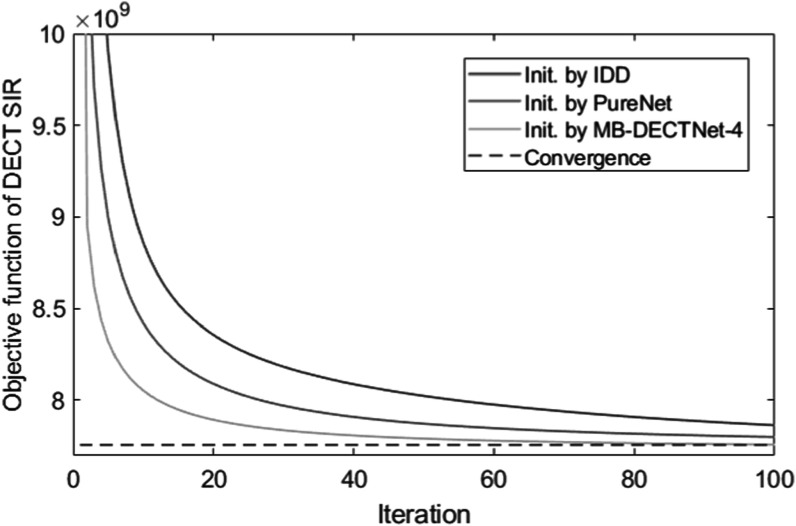
Plot of DEAM objective functions values versus iteration count.

In order to further improve the result from the neural network, we initialized the traditional DECT SIR using the deep learning-based model and compared the performance with the traditional IDD method on the same task. Specifically, we used IDD, Pure Net, and MB-DECTNet-4 as the initial condition of DEAM with 33 ordered subsets, and plotted the objective function values of DEAM versus iterations. The plot is shown in figure 9. As it is shown, DEAM initialized by MB-DECTNet-4 is close to convergence after 80 iterations, while DEAM initialized by IDD takes 100 iterations to reach the objective function value of DEAM initialized by MB-DECTNet-4 at the 20th iteration.

**Figure 10.**
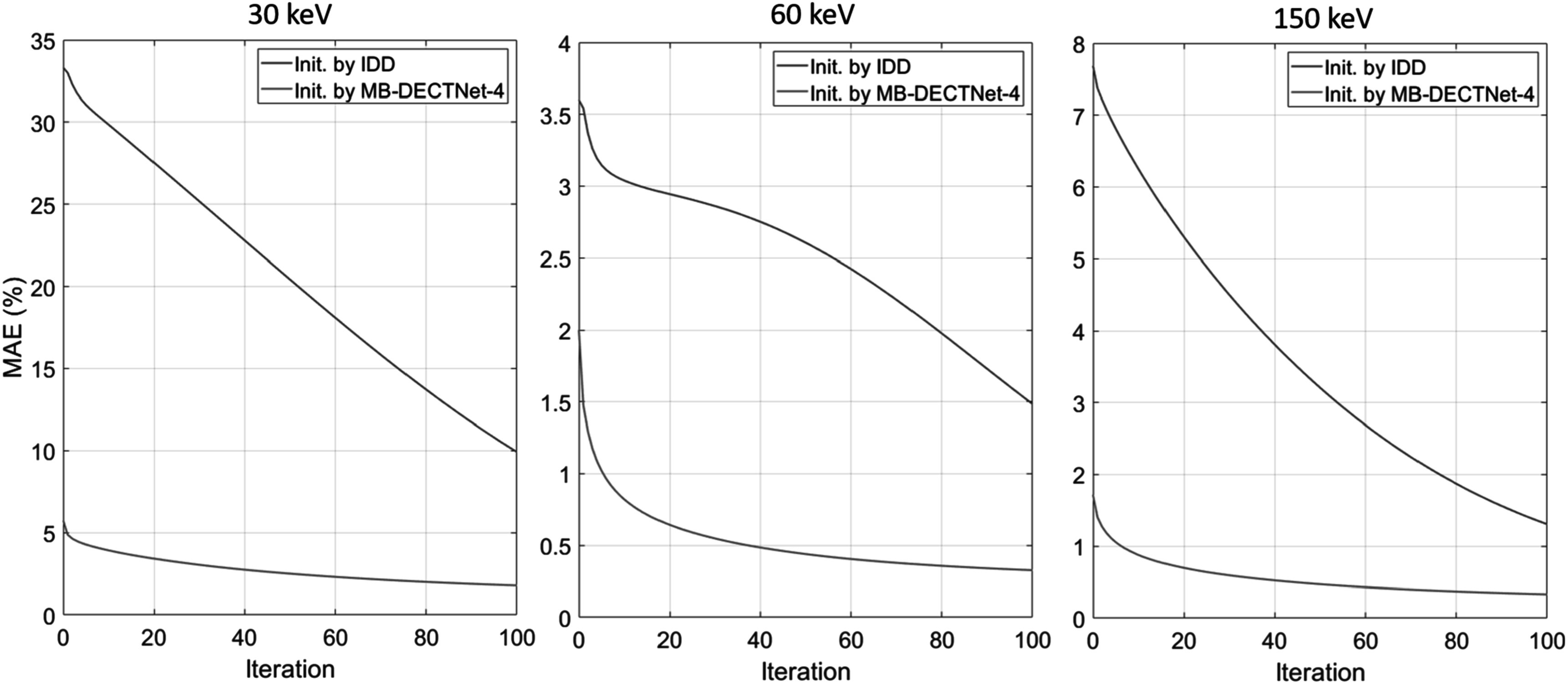
Plot of percentage MAE versus iteration.

Figure 10 shows the percentage MAE of the estimation against the reference image versus iteration, which provides another view of the convergence analysis. As we can see, MAEs of DEAM initialized by IDD start from 35%, 3.5%, and 7.5%, and gradually decrease to 10%, 1.5%, 1.4% at 30, 60, and 150 keV, respectively, while DEAM initialized by MB-DECTNet-4 reaches 1% MAE at the 8th iteration for 60 and 150 keV. The errors at 30 keV are significantly larger than the errors at 60 and 150 keV. This disparity arises primarily because very few photons with energies near 30 keV are detected. Additionally, the low- and high-energy spectra greatly down-weight the contribution of the modeled LAC at extremely low energies.

## 4. Conclusions and discussion

In this work, we proposed a model-based neural network with 3D U-Net as the backbone for accurate DECT reconstruction and showed the feasibility of the approach. To the best of our knowledge, it is the first time that a model-based unrolling is developed and trained end-to-end to estimate basis components from dual-energy CT sinograms. It is worth noting that our network parameters can be different for different update blocks since we observed that the proposed network with arbitrary weights outperformed the same network with the weights forced to the same across blocks.

Our proposed MB-DECTNet is capable of reducing noise and increasing resolution, and can be incorporated with numerous joint statistical DECT algorithms. Trained with a relatively small dataset, the quantitative results show that the proposed method with 4 update blocks can achieve 2% MAE at 60 and 150 keV on basis components estimation from clinically acquired DECT measurements, and significantly outperformed the pure deep-learning baseline. The performance of MB-DECTNet becomes better with the increase of the number of update blocks from 1 to 4, though they have comparable numbers of trainable parameters. This agrees with our intuition since the DC layer in each update block provides the network with additional information regarding the discrepancy between the estimation and the measurement.

However, due to limitations in available GPU resources, we were unable to test the proposed method with more update blocks. Accommodating additional update blocks would require reducing the model size, resulting in an insufficient number of trainable parameters for each individual network and significantly longer training times. In future studies, deep equilibrium architectures can be explored to simulate an infinite number of iterations and learn the stationary point of deep unrolling by using the output of the update block as the input (Gilton *et al* 2021).

We acknowledge that while the result is promising, there are still errors in certain regions that may be too large for clinical applications requiring high accuracy. Future evaluations of this approach should involve a more extensive dataset, for both training and testing. As noted above, the patient scans are collected within a human subject protocol; the results of this and related studies motivate increasing the number of patients and variety of scanners included in the protocol. The test set in the future should include scanning a diverse set of objects with known composition.

Furthermore, in our pursuit of enhancing estimation accuracy, we have demonstrated the feasibility of employing our proposed method as an initialization technique for DECT SIR. Plots of the objective function and MAE against the DECT SIR iteration reveal that DECT SIR initialized by MB-DECTNet converges five times faster than when initialized using traditional image-domain methods. After 10 iterations, DECT SIR initialized by MB-DECTNet-4 achieves subpercentage errors at 60 and 150 keV, demonstrating a notable improvement in quantitative accuracy. The total processing time for MB-DECTNet-4 (12 min) followed by 10 iterations of DEAM with 33 ordered subsets (15 min) is under 30 min.

## Data Availability

The data cannot be made publicly available upon publication due to legal restrictions preventing unrestricted public distribution. The data that support the findings of this study are available upon reasonable request from the authors.

## Appendix. Derivation of DEAM

The data fidelity term (9) in the energy domain could be decoupled as

\begin{eqnarray*}{ \mathcal I }({\boldsymbol{d}}\parallel {\boldsymbol{g}}({\boldsymbol{c}}))=\mathop{\min }\limits_{p}\displaystyle \sum _{j}\displaystyle \sum _{y}\displaystyle \sum _{E}\left({p}_{j}(y,E)\mathrm{ln}\displaystyle \frac{{p}_{j}(y,E)}{{q}_{j}(y,E)}-{p}_{j}(y,E)+{q}_{j}(y,E)\right)\end{eqnarray*}

\begin{eqnarray*}{\mathrm{s}}.{\mathrm{t}}.\quad \displaystyle \sum _{E}{p}_{j}(y,E)={d}_{j}(y)\quad \forall j,y\end{eqnarray*}

where

\begin{eqnarray*}{q}_{j}(y,E)={I}_{0,j}(y,E){e}^{-{\sum }_{x}h(x,y){\sum }_{i}{c}_{i}(x){\mu }_{i}(E)}.\end{eqnarray*}

Solving the Lagrangian yields

\begin{eqnarray*}{p}_{j}(y,E)={q}_{j}(y,E)\displaystyle \frac{{d}_{j}(y)}{{g}_{j}(y)}.\end{eqnarray*}

Consequently, minimizing the I-divergence is equivalent to alternately estimating *p*_*j*_(*y*, *E*) and *q*_*j*_(*y*, *E*) subject to the constraints *c*_*i*_(*x*) ≥ 0 for all *i* and *x*, i.e.

\begin{eqnarray*}c=\arg \,{\min }_{c}\displaystyle \sum _{{\mathrm{j}}}\displaystyle \sum _{{\mathrm{y}}}\displaystyle \sum _{{\mathrm{E}}}\left({\hat{p}}_{j}({\mathrm{y}},{\mathrm{E}})\mathrm{ln}\displaystyle \frac{{\hat{p}}_{j}({\mathrm{y}},{\mathrm{E}})}{{{\mathrm{q}}}_{j}({\mathrm{y}},{\mathrm{E}}:{\mathrm{c}})}-{\hat{p}}_{j}({\mathrm{y}},{\mathrm{E}})+{{\mathrm{q}}}_{j}({\mathrm{y}},{\mathrm{E}}:{\mathrm{c}})\right)\end{eqnarray*}

\begin{eqnarray*}{q}_{j}(y,E)={I}_{0,j}(y,E){e}^{-{\sum }_{x}h(x,y){\sum }_{i}{\hat{c}}_{i}(x){\mu }_{i}(E).}\end{eqnarray*}

\begin{eqnarray*}{p}_{j}(y,E)={\hat{q}}_{j}(y,E)\displaystyle \frac{{d}_{j}(y)}{{\hat{g}}_{j}(y)}.\end{eqnarray*}

Equation (A5) may be further decoupled as

\begin{eqnarray*}\displaystyle \sum _{j}{\sum }_{y}{\sum }_{E}\left({\hat{p}}_{j}(y,E){\sum }_{x}h(x,y){\sum }_{i}{\mu }_{i}(E){c}_{i}(x)+{I}_{0,j}(y,E){e}^{-{\sum }_{x}h(x,y){\sum }_{i}{c}_{i}(x){\mu }_{i}(E)}\right)\end{eqnarray*}

\begin{eqnarray*}\begin{array}{l}=\displaystyle \sum _{j}\displaystyle \sum _{y}\displaystyle \sum _{E}\left({\hat{p}}_{j}(y,E)\displaystyle \sum _{x}h(x,y)\displaystyle \sum _{i}{\mu }_{i}(E){c}_{i}(x)\right.\\ \quad \left.+{\hat{q}}_{j}(y,E){e}^{-\displaystyle \sum _{x}h(x,y)\displaystyle \sum _{i}\left({\hat{c}}_{i}(x)-{c}_{i}(x)\right){\mu }_{i}(E)}\right)\end{array}\end{eqnarray*}

\begin{eqnarray*}\begin{array}{l}\leqslant \displaystyle \sum _{j}\displaystyle \sum _{y}\displaystyle \sum _{E}\displaystyle \sum _{x}\displaystyle \sum _{i}\left(\Space{0ex}{3.45ex}{0ex}{\hat{p}}_{j}(y,E)h(x,y){\mu }_{i}(E){c}_{i}(x)\right.\\ \quad \left.+\displaystyle \frac{1}{{Z}_{1}(i){Z}_{2}(x)}{\hat{q}}_{j}(y,E)h(x,y){\mu }_{i}(E){e}^{{Z}_{1}(i){Z}_{2}(x)\left({\hat{c}}_{i}(x)-{c}_{i}(x)\right)}\right).\end{array}\end{eqnarray*}

This spatially decoupled surrogate objective function allows parallel updating of all the voxels after the back-projection of ${\hat{p}}_{j}(y,E){\mu }_{i}(E)$ and ${\hat{q}}_{j}(y,E){\mu }_{i}(E)$, so the update procedure could be super fast if multi-thread computing is enabled. Equation (A9) is derived by simply substituting

\begin{eqnarray*}{I}_{0,j}(y,E)={\hat{q}}_{j}(y,E)/{e}^{-{\sum }_{x}h(x,y){\sum }_{i}{\hat{c}}_{i}(x){\mu }_{i}(E)}\end{eqnarray*}

into (A8), where ${\hat{c}}_{i}(x)$ and ${\hat{q}}_{j}(y,E)$ denote the previous estimates. Equation (A10) is derived from Jensen’s inequality by setting the weighting factor to be *h*(*x*, *y*)*μ*_*i*_(*E*)/(*Z*_1_(*i*)*Z*_2_(*x*)), where *Z*_1_(*i*) and *Z*_2_(*x*) need to satisfy (Zhang 2018)

\begin{eqnarray*}\displaystyle \sum _{i}\displaystyle \frac{{\mu }_{i}(E)}{{Z}_{1}(i)}\leqslant 1\quad \forall i\qquad \mathrm{and}\qquad \displaystyle \sum _{x}\displaystyle \frac{h(x,y)}{{Z}_{2}(x)}\leqslant 1\quad \forall y.\end{eqnarray*}

Solving the first-order necessary condition of (A10) yields

\begin{eqnarray*}{c}_{i}(x)={\hat{c}}_{i}(x)-\displaystyle \frac{1}{{Z}_{i}(x)}\mathrm{log}\displaystyle \frac{{\sum }_{j}{\sum }_{y}{\sum }_{E}{\mu }_{i}(E)h(x,y){p}_{j}^{k}(y,E)}{{\sum }_{j}{\sum }_{y}{\sum }_{E}{\mu }_{i}(E)h(x,y){q}_{j}^{k}(y,E)},\end{eqnarray*}

where

\begin{eqnarray*}{q}_{j}^{k}(y,E)={I}_{0,j}\exp \left(-\displaystyle \sum _{x}h(x,y)\displaystyle \sum _{i}{\mu }_{i}(E){c}_{i}^{k}(x)\right)\end{eqnarray*}

\begin{eqnarray*}{p}_{j}^{k}(y,E)={q}_{j}^{k}(y,E)\displaystyle \frac{{d}_{j}(y)}{{\sum }_{E^{\prime} }{q}_{j}^{k}(y,E^{\prime} )}.\end{eqnarray*}
